# Peer Review of “A Local Community-Based Social Network for Mental Health and Well-being (Quokka): Exploratory Feasibility Study”

**DOI:** 10.2196/33928

**Published:** 2021-10-27

**Authors:** Ziyou Ren

**Affiliations:** 1 Center for Research Informatics University of Chicago Knapp Center for Biomedical Discovery Chicago, IL United States

**Keywords:** local social network, community health, well-being, digital health, consumer health

*This is a peer-review report submitted for the paper “A Local Community-Based Social Network for Mental Health and Well-being (Quokka): Exploratory Feasibility Study.”*

## Round 1 Review

### General Comments

The authors [1] tried to investigate the effects of a well-being theme (ie, Quokka) through setting challenges in 4 different university campuses. There were 277 participants. The author found the participants preferred local activities to remote, but there was not enough evidence to support other significant differences.

Although the author focused on an interesting topic, most of the analysis was descriptive and lacked depth. For example, the relationship between the major outcomes and well-being was not clear. Is there any measurement for mental health, such as anxiety or depression, after using Quokka? Furthermore, I was confused whether the manuscript is about Quokka, the platform, or is an intervention study using Quokka. I would appreciate if the authors could add more details to the Quokka platform if this is original. Who developed the platform? If the Quokka platform was developed by someone else, please include the reference. The conclusion and generalization of the manuscript is limited. More details can be found in the minor comments below.

### Minor Comments

1. The number of users who completed the final check-in is very different from the number after the first week. Do the authors have an explanation for the low participant rate at the final week? Would the results be any different if the authors considered using the participants at the final week as the evaluation group?

2. Was there an appropriate control group for the study? For example, would there be any survey before using Quokka?

3. Please add the author list and their affiliations to the manuscript.

4. Please provide institutional review board approval from each of the universities.

5. Please indicate when the experiment was conducted. I wonder if the results are affected by the current pandemic.

## Round 2 Review

### General Comments

This paper is a revised manuscript discussing Quokka and its application in the community. Overall, I feel the quality of manuscript improved significantly after the revision, and my comments are addressed well. I do not have any further comments.
